# Wood’s Medical and Surgical Monographs

**Published:** 1889-06

**Authors:** 


**Wood's Medical and Surgical Monographs.** Vol. II., No. 2, May, 1889 Published monthly; $10.00 a year; single copies, $1.00. William Wood & Co., New York: 56 and 58 Lafayette place.

The first article in this number, on the Preventive Treatment of Calculous Disease and the Use of Solvent Remedies, is by Sir Henry Thompson, than whom there is no more competent authority on diseases involving the genito-urinary tract. For some time surgeons have been asking themselves the question, "Cannot something more be done to prevent the *formation* of stone?" They have not been satisfied with the great progress that has lately been witnessed with the lithotrite, or even the knife. These have, indeed, been great in the results they have recently accomplished, almost completely revolutionizing the practice within the past few years, and certainly lessening the formidable character of the surgery of stone. But, wonderful as they are conceded to be, they are admirable only in the line of cure.

Keeping pace with the spirit of preventive medicine that is everywhere manifest, and that has invaded even the domain of surgery, Sir Henry, voicing the thought of genito-urinary surgeons, comprehensively and scientifically asks: "Is there not a period in the history of the process which leads to the formation of renal or vesical calculus, whether in the condition of gravel, concretion, or stone, at which it might be possible to prevent the development of a considerable deposit and the necessity for mechanically removing it?"

This is the question he proposes for solution, and he devotes three lectures—about forty-six pages—to its consideration. It is clearly within the borders of unexaggerated truth to pronounce these lectures as the most satisfactory exposition of the subject that has yet appeared, and they cannot fail to instruct the reader, no matter how learned he may be, nor however competent he may consider himself.

The second part of this number consists of a Treatise on Sprains; their Consequences and Treatment, by C. W. Mansell, Moullin, M. A., M. D., F. R. C. S., England, Assistant Surgeon and Senior Demonstrator of Anatomy at the London Hospital, etc.

The author considers this subject in an introduction and sixteen chapters, comprising 213 pages altogether, and it must be confessed that it is done in a most exhaustive manner.

These injuries have usually been considered as too trifling by surgeons to warrant much treatment, and as too insignificant by patients to permit much loss of time, or to merit much attention. Indeed, the surgeon is rarely called upon at all in the majority of cases; or, if he is consulted, his directions are seldom heeded.

The fact is, that a sprain is frequently a very important injury, and many a cripple has reason to look back to a neglected sprain as the cause of his lameness or deformity.

This is not strange when we consider how difficult it is to enforce the first and most essential element in the problem of cure,—rest. Another difficulty to be overcome in the management of sprains, is the tendency to a faulty domestic treatment, which is generally little else than maltreatment. The arnica bottle and a bandage, the latter commonly applied over-snugly, are the great cure-alls in the estimation of the housewife, or officious neighbor. The medical attendant,—that kindhearted soul, the family doctor,—too often acquiesces in the faulty treatment already employed, and thus stamps the evil that is being done with the weight of authority. If our author had done nothing else than to combat the serious errors that arise in this way, he would not have written in vain. He not only strikes hard at these, but he deals telling blows that must result in much good, if his teachings are heeded as they should be.

The importance of this subject is great, because of the frequency of these injuries, and because they come within the province of every physician. No more profitable reading can be indulged in, and the volume should be found in every doctor's library.

